# A Narrative Review on Karnofsky Performance Status in Patients With Brain Tumors: Should It Be Adjusted?

**DOI:** 10.7759/cureus.117566

**Published:** 2026-10-06

**Authors:** Idalberto L Fernandez Eng, Luis A Guzman Blanco, Manuel A Guerra Guerrero, Carlos A Crespo Bao, Leydis L Marquez Lazo, Claudia Pallasa Dominguez

**Affiliations:** 1 Department of General Medicine, Independent Researcher, Boston, USA; 2 Department of Neurological Surgery, Universidad de Ciencias Médicas de Granma, Manzanillo, CUB; 3 Department of Internal Medicine, Community Medical Group, Lakeland, USA; 4 Department of Surgery, Educational Commission for Foreign Medical Graduates, Miami, USA; 5 Department of General Medicine, Independent Researcher, Vero Beach, USA; 6 Center for Discovery and Innovation (CDI), Hackensack Meridian Health, Nutley, USA

**Keywords:** brain tumors, functional status, glioblastoma, karnofsky performance status, nano scale, neuro-oncology

## Abstract

The Karnofsky Performance Status (KPS) is one of the most widely used functional scales in oncology to assess patients’ functional status, including their ability to work, perform self-care, and the need for assistance. Although its clinical utility is well established, KPS is generally applied uniformly across cancer populations, without specifically accounting for the distinct patterns of disability observed in patients with brain tumors. These patients may experience focal neurological deficits, cognitive impairment, and other neurological changes that can substantially affect functional status and may not be adequately captured by the current KPS framework.

This narrative review examines the available evidence regarding the limitations of the standard KPS in patients with primary and metastatic brain tumors and considers the potential value of adjusting or complementing its use in clinical practice and research. A non-systematic literature review was conducted between August and September 2026 in PubMed and Google Scholar, using terms including “Karnofsky Performance Status,” “KPS,” “brain metastasis,” “brain tumor,” “neuro-oncology,” “glioma,” and “radiotherapy” in various combinations. Studies published in English were considered, with priority given, when possible, to studies published within the past five years and to those comparing KPS with neurological, cognitive, or patient-reported measures of function. The reviewed literature demonstrates substantial interobserver variability in KPS assignment, with kappa coefficients as low as 0.19-0.26 in studies reporting poor agreement. Discordance was also identified between clinician-assigned KPS and objective cognitive impairment, with up to 36% of patients considered to have good KPS scores showing clinically relevant cognitive dysfunction. In addition, medical decision-making capacity may be impaired despite high KPS scores, with only 46% of patients with KPS 90-100 classified as capable using a standardized instrument. Discordance between KPS and patient-reported outcomes was also reported. These limitations have prompted the development and use of complementary tools, including the Neurologic Assessment in Neuro-Oncology (NANO) scale and brain tumor-specific assessments of instrumental activities of daily living (IADL). Overall, the KPS retains important prognostic and practical value; however, the available evidence supports complementing its use in neuro-oncology with objective neurological assessment, brief cognitive screening, and, when possible, patient- or caregiver-reported measures. Functional status should also be assessed dynamically over time, avoiding anchoring the patient’s overall functional assessment to a single KPS value, rather than using KPS as the sole and sufficient measure of functional status.

## Introduction and background

The Karnofsky Performance Status (KPS), introduced by Karnofsky and Burchenal in 1949, was originally developed as a global measure of functional impairment in patients with cancer. It consists of an 11-point scale ranging from 100, representing normal functioning with no evidence of disease, to 0, representing death, in 10-point increments. The original criteria describe three main levels of independence: patients who are able to perform normal activities and work without special care (KPS 80-100), those who are unable to work but can live at home and take care of most of their personal needs with different levels of assistance (KPS 50-70), and those who are unable to care for themselves and require institutional or hospital care (KPS 10-40) [1]. Since its introduction, the KPS has become widely used for prognostic assessment and treatment decisions and especially as an eligibility criterion for oncology clinical trials, where a KPS ≥ 70 is commonly required. This threshold is clinically relevant because patients with borderline functional status may be excluded from potentially beneficial treatments or clinical trials based solely on their KPS score.

In neuro-oncology, the KPS has generally been used in the same way as in other types of cancer, without specific adjustments for patients with brain tumors. However, primary and metastatic brain tumors often produce a different pattern of disability compared with systemic cancer. Patients may develop focal neurological deficits, such as hemiparesis, aphasia, or visual field disturbances, as well as cognitive impairment affecting attention, memory, or executive function, and changes in behavior or personality. These problems may occur even when a patient’s physical ability to perform basic self-care remains relatively preserved, or may result in isolated functional limitations in patients who are otherwise cognitively intact. The KPS focuses mainly on the ability to work and perform basic self-care and does not include specific items to capture these neurological and cognitive domains, which are particularly relevant in this population [2,3].

Although limitations of the KPS in neuro-oncology have been mentioned over the years in different studies, case reports, conference abstracts, and papers focusing on specific areas such as decision-making capacity or quality of life, these findings have remained fragmented in the literature. To our knowledge, no comprehensive narrative review has brought this evidence together to provide a clearer understanding of how KPS performs in patients with brain tumors and whether its use should be adjusted for this population. This review aims to address this gap.

The objective of this narrative review is to examine the available evidence on the limitations of the standard KPS when applied to patients with brain tumors and, based on this evidence, to support adjusting or systematically complementing its use in both clinical practice and clinical trial design, rather than continuing to use it as the sole and sufficient measure of functional status in neuro-oncology.

Methods

A narrative (non-systematic) literature search was conducted between August and September 2026 in PubMed and Google Scholar, using keywords including “Karnofsky Performance Status,” “KPS,” “brain metastasis,” “brain tumor,” “neuro-oncology,” “glioma,” and “radiotherapy,” in various combinations. Studies published in English were considered, with priority given, when possible, to studies published within the past five years and to those comparing KPS with neurological, cognitive, or patient-reported measures of function. Case reports, letters to the editor, and animal studies were excluded. Because this was a narrative, non-systematic review, the search was not intended to provide a systematically exhaustive identification of all available evidence.

## Review

What the KPS measures and does not measure

The KPS was originally developed in the context of cancer treatment to standardize the assessment of patients’ functional status before treatment [1]. The scale mainly evaluates overall functional independence based on the ability to work, perform usual activities, and care for oneself, and the level of assistance required. Although this approach is clinically practical, it has important limitations when applied to patients with brain tumors. The KPS does not include separate domains to specifically assess cognition, language, visual fields, or behavior. These deficits may influence the KPS when they affect a patient’s ability to perform daily activities or care for themselves, but their presence and severity are not directly characterized by the scale. As a result, patients with very different neurological profiles may receive similar KPS scores when they have a similar overall level of independence. This distinction is particularly relevant in neuro-oncology, where focal neurological or cognitive dysfunction may substantially affect a patient’s functional status even when overall physical condition and basic self-care abilities are relatively preserved [2,3].

Interobserver variability

A second limitation is that assigning a KPS score requires some degree of interpretation by the evaluator. Methodological studies have shown that its reliability can improve when more explicit scoring criteria and trained evaluators are used, suggesting that part of the variability may depend on how the scale is applied [4,5]. In a systematic review of 15 studies, Chow et al. found heterogeneous results regarding interobserver reliability: three studies reported poor agreement (kappa: 0.19-0.26), four moderate agreement (kappa: 0.31-0.72), and six high agreement. In addition, four studies found higher reliability for the KPS compared with the Eastern Cooperative Oncology Group Performance Status (ECOG-PS) and Palliative Performance Scale (PPS) [6]. Therefore, the reproducibility of the KPS appears to depend, at least in part, on the observers, clinical setting, and way in which the scale is applied.

This variability may have important implications in neuro-oncology. Frappaz et al. found that KPS scores varied significantly according to the evaluator’s age, specialty, and experience, with residents and general physicians assigning lower scores than oncologists. These differences could determine whether patients reached the KPS ≥ 70 threshold, which is frequently used as an eligibility criterion for clinical trials [7]. Similarly, Panciroli et al. found only fair overall agreement among oncologists, investigators, patients, and caregivers (Fleiss’ κ = 0.354), with particularly low agreement between oncologists and patients (κ = 0.250) [8]. These findings suggest that KPS may reflect not only the patient’s functional impairment but also, to some extent, the perspective and experience of the person assigning the score.

Difficulty distinguishing focal neurological deficits from global functional decline

Another important limitation is the difficulty of distinguishing focal neurological deficits from overall clinical deterioration. Patients with hemiparesis, aphasia, or visual field loss may have significant limitations in specific activities while maintaining preserved cognition, judgment, communication, systemic stability, and sufficient physiological reserve to tolerate oncologic treatment. Because the KPS places considerable emphasis on activity, self-care, and the need for assistance, a focal neurological deficit may have a substantial effect on the score and may not fully reflect the patient’s overall clinical condition.

Frappaz et al. demonstrated the clinical relevance of this limitation by comparing KPS and the World Health Organization Performance Status (WHO-PS) across standardized brain tumor scenarios. Agreement between the two scales decreased as neurological disability increased, with discordance affecting trial eligibility in approximately 16%-46% of the more impaired scenarios. In nearly all discordant cases, the KPS would have excluded patients who would have been considered eligible based on the WHO-PS, with hemiplegia leading to greater exclusion than expressive aphasia. These findings suggest that physically disabling focal deficits may have a disproportionate effect on KPS classification and should not necessarily be interpreted as equivalent to generalized clinical deterioration [9].

KPS insensitivity to cognitive impairment

Cognitive impairment, together with focal neurological deficits, is one of the most relevant domains in patients with brain tumors and one that is not directly assessed by the KPS. This gap can produce a discrepancy between the global functional independence reflected by the KPS and the patient’s true cognitive status. In a study of 273 patients with brain tumors, Panzer et al. found that clinician-assigned KPS did not correlate significantly with objective Montreal Cognitive Assessment (MoCA) scores in the evaluated subgroup and that up to 36% of patients considered to have good functional status showed clinically relevant cognitive dysfunction, whether objective or self-perceived [10]. This discrepancy can also have concrete clinical consequences. Martin et al. assessed the capacity to give informed consent in 71 patients with malignant brain tumors using the Capacity to Consent to Treatment Instrument (CCTI) and found that only 46% of patients with KPS 90-100, considered to have minimal disability on the scale, were classified as capable; this proportion decreased to 23% among those with KPS 70-80 and to 0% in the KPS 50-60 group [11]. These findings show that a high KPS does not necessarily imply preserved cognitive function or decision-making capacity, which may have implications for informed consent, advance care planning, and patient selection for clinical research. Similarly, Gerstenecker et al. found that cognitive impairment was frequent in patients with brain metastases despite a relatively preserved functional status, with a median KPS of 80. Deficits were identified in memory, attention, executive function, and language, reinforcing the possible discrepancy between global functional performance as assessed by the KPS and underlying cognitive function [12].

Discordance between KPS and patient-reported outcomes

As early as 1992, in one of the first studies of quality of life in neuro-oncology, Mackworth et al. administered a quality of life questionnaire to 200 patients with brain tumors and compared the results with KPS scores. They found that, among patients with KPS 90-100, who represented approximately two-thirds of the sample, the scale was “difficult to interpret,” whereas the questionnaire provided a more detailed assessment of the patients’ condition. In addition, depression, which was present in approximately half of the patients, was not predicted by the KPS [13]. More recently, Agarwal et al. compared, in 140 patients with brain metastases from lung cancer, a patient-reported functional score derived from the physical and role functioning subscales of the European Organisation for Research and Treatment of Cancer - Quality of Life Questionnaire - Core 30 (EORTC QLQ-C30) with physician-assigned KPS. They found only modest agreement between the two measures (polychoric correlation = 0.46). The patient-reported score retained independent prognostic value for survival and showed better model fit (adequacy index: 95.2% versus 85.6% for KPS), whereas KPS lost prognostic significance when both variables were included in the same model [14]. Overall, these findings suggest that functional assessment based solely on KPS may not fully reflect the patient’s functional experience and quality of life and that patient-reported outcomes may provide clinically relevant complementary information.

KPS insensitivity to instrumental activities of daily living

The cognitive impairments described in the previous section can also affect a patient’s capacity to function independently [15]. Basic activities of daily living comprise self-care tasks, whereas instrumental activities of daily living (IADL) involve more complex tasks such as managing money, organizing medication, or handling everyday affairs. The Barthel Index assesses basic activities. The KPS, by contrast, summarizes overall functioning, including self-care, work, and usual activities. However, this global assessment does not separately describe difficulties in specific instrumental tasks. Understanding these difficulties is necessary to evaluate a patient’s autonomy [16,17].

This need motivated the development of the EORTC IADL-BN32. Oort et al. found that a questionnaire designed for people with dementia did not cover the full range of everyday difficulties experienced by patients with brain tumors. The resulting disease-specific instrument contains 32 items across five dimensions: household activities, activities requiring sustained concentration, use of devices and communication skills, administrative tasks, and social activities. It also includes two individual items on work performance and medication management. Its content allows for a more detailed description of everyday functioning than that offered by a global functional score [16].

The 2024 international validation study included 326 patients with gliomas or brain metastases, assessed at baseline, two weeks, and three months. The scales showed good to excellent internal consistency, with Cronbach’s alpha of 0.86-0.97. Their scores also differentiated patients with and without neurocognitive impairments or complaints, supporting construct validity. The study included the KPS and the Barthel Index as functional comparators. These results support the use of a disease-specific IADL measure as a complement to the KPS rather than a replacement: the instrument did not demonstrate superiority over the KPS, but it provides additional information on everyday tasks that the global score does not capture. Limitations remain regarding the heterogeneity of neurocognitive assessment, cross-cultural validity, and sensitivity to change [16].

The Neurologic Assessment in Neuro-Oncology (NANO) scale

The assessment of everyday activities can be complemented by a standardized description of neurological status. With this aim, Nayak et al. introduced the NANO scale in 2017. Its development addressed the need to quantify clinically relevant neurological findings during consultation and refine the clinical component of response assessment. Through direct observation and examination, NANO evaluates nine domains: gait, strength, upper limb ataxia, sensation, visual fields, facial strength, language, level of consciousness, and behavior. Each domain has defined levels of impairment, facilitating the recording of specific changes during follow-up [17].

The initial evaluation included 220 patients from eight hospitals. Interobserver agreement exceeded 90% in every domain, with calculated kappa values ranging from 0.35 to 0.83. This apparent discrepancy between high percent agreement and more modest kappa values reflects the low prevalence of abnormal findings in several domains, which lowers kappa even when the observed agreement is high. Agreement was uneven: it was lowest for behavior and highest for language; kappa could not be calculated for consciousness owing to the scarcity of abnormal observations. Median administration time was 4 minutes (interquartile range: 3-5) [17]. As subsequent evidence, the Indonesian adaptation published by Angelita et al. in 2026 reported kappa values of 0.629-0.935 [18]. However, its 30 participants and single-center design limit the generalizability of these results.

NANO and IADL questionnaires provide different and complementary information. While the latter describe difficulties in complex everyday tasks, NANO characterizes neurological findings, and the KPS/Eastern Cooperative Oncology Group (ECOG) summarizes overall functioning. The original NANO study proposed combining this assessment with the radiologic component of the Response Assessment in Neuro-Oncology (RANO) criteria, without replacing cognitive testing or patient-reported outcomes [17]. NANO therefore does not, on its own, resolve the assessment of instrumental autonomy. This distinction supports an evaluation approach that brings together global functioning, everyday performance, and neurological status, and sets up the discussion of its practical implications.

Historical precedent for complementing KPS

The recognition that KPS alone may be insufficient for prognostic stratification in brain tumors is not new. Since the 1990s, Radiation Therapy Oncology Group (RTOG) recursive partitioning analysis (RPA) classifications have combined KPS with additional clinical variables to improve prognostic discrimination. In malignant gliomas and glioblastoma, these models incorporated factors such as age, extent of resection, and neurological function alongside KPS [19,20], while the RPA for brain metastases incorporated age and extracranial disease status [21]. Although these models were not designed to modify KPS itself, their long-standing use illustrates that KPS provides more meaningful information when interpreted together with other clinical characteristics. More recently, studies have demonstrated discordance between KPS and neurological function, and have supported the use of complementary tools such as the NANO scale for more objective assessment of neurological function [9,17]. Together, these findings provide a historical and contemporary rationale for complementing KPS rather than relying on it as a standalone measure in neuro-oncology.

Why not simply replace KPS?

These limitations, however, do not justify abandoning KPS. Its extensive historical use provides valuable prognostic information and allows comparison across decades of oncologic and neuro-oncologic literature. Sasaki et al. demonstrated in patients with glioblastoma that KPS at hospital discharge and improvement in KPS during hospitalization were associated with prognosis, emphasizing that performance status is dynamic and that a single low value during an acute or postoperative period may not adequately represent the patient’s subsequent functional trajectory [22]. This supports interpreting KPS in the clinical context and over time rather than treating an individual score as a fixed representation of the patient’s functional status. The main limitations of KPS identified across the reviewed literature, together with the corresponding evidence from individual studies, are summarized in Table 1.

**Table 1:** Summary of Key Evidence on KPS Limitations in Neuro-Oncology KPS: Karnofsky Performance Status, ECOG-PS: Eastern Cooperative Oncology Group Performance Status, PPS: Palliative Performance Scale, WHO-PS: World Health Organization Performance Status, MoCA: Montreal Cognitive Assessment, CCTI: Capacity to Consent to Treatment Instrument, PRO: patient-reported outcome

Study	Population/design	Comparison	Key finding
Chow et al. [6]	Systematic review, 15 studies	KPS interobserver reliability	3 studies poor agreement, 4 moderate, 6 high; KPS more reliable than ECOG-PS/PPS in 4 studies
Panciroli et al. [8]	Oncologists, investigators, patients, caregivers	Interobserver agreement	Fleiss’ κ = 0.354 overall; κ = 0.250 between oncologists and patients
Frappaz et al. [9]	Standardized brain tumor scenarios	KPS versus WHO-PS	Discordance in 16%-46% of more impaired scenarios; KPS excluded patients who were WHO-PS-eligible
Panzer et al. [10]	273 patients with brain tumor	Clinician-assigned KPS versus MoCA	No significant correlation; 36% with good KPS had clinically relevant cognitive dysfunction
Martin et al. [11]	71 patients with malignant brain tumors	KPS versus decision-making capacity (CCTI)	Capable: 46% (KPS 90-100), 23% (KPS 70-80), 0% (KPS 50-60)
Gerstenecker et al. [12]	Patients with brain metastases	KPS versus cognitive function	Frequent cognitive impairment despite median KPS of 80
Mackworth et al. [13]	200 patients with brain tumors	KPS versus quality of life questionnaire	KPS “difficult to interpret” at KPS 90-100; did not predict depression
Agarwal et al. [14]	140 patients with brain metastases (lung cancer)	KPS versus patient-reported outcome (EORTC QLQ-C30)	Polychoric correlation = 0.46; PRO showed better prognostic model fit (95.2% versus 85.6%)
Sasaki et al. [22]	Patients with glioblastoma	KPS at discharge and change in KPS	Both associated with prognosis; supports dynamic assessment

Additionally, two of the studies informing key findings in this review were available only as conference abstracts [7,10]. This limited the methodological and outcome information available for evaluation and should be considered when interpreting their findings, including the reported association between KPS and cognitive impairment.

Contemporary neuro-oncology therefore supports a multidimensional rather than replacement-based approach. European Association of Neuro-Oncology (EANO) guidelines recommend considering KPS together with neurological function, age, and individual risks and benefits when making treatment decisions in diffuse glioma [23]. Similarly, the NANO scale provides a complementary, domain-specific assessment of neurological function that a global performance score cannot capture [17]. Thus, the most defensible strategy is refinement rather than replacement: KPS should retain its prognostic and comparative role while being interpreted longitudinally and complemented by structured neurological assessment when focal or potentially reversible deficits are present.

Practical implications

The evidence reviewed has several practical implications for neuro-oncology. A high KPS should not be assumed to indicate preserved cognitive function or decision-making capacity, while a low KPS should not necessarily be interpreted as permanent or generalized functional decline. Patients with focal neurological deficits or acute postoperative complications may receive a low KPS despite having preserved cognition, systemic stability, and potential for functional recovery. Importantly, Sasaki et al. demonstrated that both KPS at hospital discharge and improvement in KPS during hospitalization were associated with prognosis in patients with glioblastoma, supporting the importance of evaluating functional status dynamically rather than relying on a single score [22].

This principle may be particularly relevant for clinical trial eligibility, where rigid KPS thresholds can exclude patients whose functional status may improve with recovery or treatment. Rather than using KPS as an absolute cutoff, eligibility assessment could incorporate the clinical context, neurological function, and longitudinal changes in performance status. Future prospective multicenter studies should evaluate whether dynamic KPS assessment, complemented when appropriate by neurological, cognitive, and patient-reported measures, can improve patient selection and functional characterization. The goal is not to replace KPS, but to avoid anchoring clinical decisions to a single value and instead provide a more complete and dynamic assessment of the patient’s functional trajectory [17,22,23].

## Conclusions

Overall, the KPS remains a useful tool for the global assessment of functional status, and its prognostic and historical value remains well established. However, the evidence reviewed in this narrative review demonstrates that its broad functional criteria and lack of specific neurological and cognitive domains limit its ability to provide a complete assessment of patients with brain tumors when used alone. These limitations support greater standardization of KPS assessment and its systematic complementation with tools such as the NANO scale, brief cognitive screening, and patient- or caregiver-reported measures, rather than abandoning its use.

We do not propose abandoning the KPS, as its prognostic and historical value remains well established. Rather, it should not be considered the sole and sufficient measure of functional status in neuro-oncology. Prospective multicenter studies are needed to formally evaluate combined approaches, such as KPS plus NANO, cognitive screening, and patient-reported measures, and to determine how these assessments can be standardized in routine clinical practice and eligibility criteria for neuro-oncology clinical trials.

## References

[1] Karnofsky DA, Burchenal JH (1949). The clinical evaluation of chemotherapeutic agents in cancer. Evaluation of Chemotherapeutic Agents.

[2] Samman RR, Timraz JH, Mosalem Al-Nakhli A (2024). The impact of brain tumors on emotional and behavioral functioning. Cureus.

[3] Parsons MW, Dietrich J (2021). Assessment and management of cognitive symptoms in patients with brain tumors. Am Soc Clin Oncol Educ Book.

[4] Schag CC, Heinrich RL, Ganz PA (1984). Karnofsky performance status revisited: reliability, validity, and guidelines. J Clin Oncol.

[5] Mor V, Laliberte L, Morris JN, Wiemann M (1984). The Karnofsky Performance Status Scale. An examination of its reliability and validity in a research setting. Cancer.

[6] Chow R, Chiu N, Bruera E (2016). Inter-rater reliability in performance status assessment among health care professionals: a systematic review. Ann Palliat Med.

[7] Frappaz D, Taillandier L, Levard-Bonneville A, Sore J, Ricard D, Schiffler C, Weller M (2018). P05.28 Karnofsky performance score of brain tumor patients depends on clinician status. Neuro Oncol.

[8] Panciroli C, Estival A, Lucente G (2017). ECOG or Karnofsky performance status to assess functionality in glioblastoma patients among different observers. J Mol Biomark Diagn.

[9] Frappaz D, Bonneville-Levard A, Ricard D, Carrie S, Schiffler C, Xuan KH, Weller M (2021). Assessment of Karnofsky (KPS) and WHO (WHO-PS) performance scores in brain tumour patients: the role of clinician bias. Support Care Cancer.

[10] Panzer M, Vera E, Acquaye A (2021). NCOG-47. Congruence between provider reported performance status and both objective and perceived cognition in a glioma population. Neuro Oncol.

[11] Martin RC, Gerstenecker A, Nabors LB, Marson DC, Triebel KL (2015). Impairment of medical decisional capacity in relation to Karnofsky Performance Status in adults with malignant brain tumor. Neurooncol Pract.

[12] Gerstenecker A, Nabors LB, Meneses K (2014). Cognition in patients with newly diagnosed brain metastasis: profiles and implications. J Neurooncol.

[13] Mackworth N, Fobair P, Prados MD (1992). Quality of life self-reports from 200 brain tumor patients: comparisons with Karnofsky performance scores. J Neurooncol.

[14] Agarwal JP, Chakraborty S, Laskar SG (2017). Prognostic value of a patient-reported functional score versus physician-reported Karnofsky Performance Status Score in brain metastases. Ecancermedicalscience.

[15] Oort Q, Dirven L, Sikkes SA (2022). Do neurocognitive impairments explain the differences between brain tumor patients and their proxies when assessing the patient's IADL?. Neurooncol Pract.

[16] Oort Q, Reijneveld JC, Sikkes SA (2024). Instrumental activities of daily living in neuro-oncology: international validation of the EORTC IADL-BN32 questionnaire. Eur J Cancer.

[17] Nayak L, DeAngelis LM, Brandes AA (2017). The Neurologic Assessment in Neuro-Oncology (NANO) scale: a tool to assess neurologic function for integration into the Response Assessment in Neuro-Oncology (RANO) criteria. Neuro Oncol.

[18] Angelita MC, Andini PW, Andriani R, Sofyan HR, Savitri I, Aninditha T (2026). Translation and validation of the Neurologic Assessment in Neuro-Oncology scale into Indonesian (Nano-Ina). Asian Pac J Cancer Prev.

[19] Curran WJ Jr, Scott CB, Horton J (1993). Recursive partitioning analysis of prognostic factors in three Radiation Therapy Oncology Group malignant glioma trials. J Natl Cancer Inst.

[20] Li J, Wang M, Won M, Shaw EG, Coughlin C, Curran WJ Jr, Mehta MP (2011). Validation and simplification of the Radiation Therapy Oncology Group recursive partitioning analysis classification for glioblastoma. Int J Radiat Oncol Biol Phys.

[21] Gaspar L, Scott C, Rotman M (1997). Recursive partitioning analysis (RPA) of prognostic factors in three Radiation Therapy Oncology Group (RTOG) brain metastases trials. Int J Radiat Oncol Biol Phys.

[22] Sasaki S, Tsukamoto S, Ishida Y (2024). The Karnofsky Performance Status at discharge is a prognostic indicator of life expectancy in patients with glioblastoma. Cureus.

[23] Weller M, van den Bent M, Preusser M (2021). EANO guidelines on the diagnosis and treatment of diffuse gliomas of adulthood. Nat Rev Clin Oncol.

